# Dr. Wilson, on Opium

**Published:** 1804-02-01

**Authors:** C. Philips Wilson

**Affiliations:** Worcester


### *To the Editors of the Medical and Physical Journal.*

GENTLEMEN;

THE chief source of dispute respecting the action of opium on living animals has been the question, whether it is a stimulus or a sedative? On the subject of stimulus and sedative so much has been written, that it seems at first sight that nothing new can be said on it. If, however, the attention be directed rather to the phenomena of animal life than to prevalent opinions, we shall, I think, be led to conclusions in some respects different from those hitherto advanced.

By stimulus is meant that which excites, by sedative that which depresses, the powers of life. The late Doctor Brown maintained, that the idea of a sedative is unfounded; and that wherever a depression of the powers of life takes place, it is the consequence either of the abstraction of stimuli or excitement. But has not death been the immediate consequence of a large quantity of spirit of wine suddenly received into the stomach, of some of the passions, &c? We also find loss of power, without previous excitement, the consequence of agents whose action is confined to, or chiefly exerted on, the muscular system. When a considerable quantity of an infusion of opium or tobacco is thrown into the heart of a frog, it does not excite violent contractions followed by a loss of excitability, but instant paralysis.

Laying aside Dr. Brown's hypothesis, shall we find the prevalent opinion, which divides all agents into stimuli and sedative, more consistent with observation? We see the foregoing agents, the passions of the mind, opium, tobacco, &c. acting as sedatives; where then are we to look for stimuli? To the same agents; for all of them, if applied in a less degree or quantity, excite the powers of life. A small quantity of spirit of wine received into the stomach, occasions excitement. There is no passion which may not act as a stimulus; fear itself, when not in such a degree as to overcome the powers of life, gives temporary vigour. It is unnecessary to allude to the numberless experiments and observations, which prove that opium and tobacco, applied in small quantity, occasion excitement.

The same, I believe, will be found true of every other agent. Applied to a certain extent, they all excite the powers of life; beyond this they depress or destroy them.

If so, we cannot regard any agent as merely stimulant or sedative. It is true that some possess more of the stimulant, others of the sedative power; but all seem capable of producing both effects. And that the latter effect is independent of the former appears from this, that the one bears no particular proportion to the other, which, were the sedative a consequence of the stimulant operation, would be the case.

Opium then, like other agents, acts both as a stimulus and a sedative, according to the quantity applied; and this seems to be a principal cause of the difference of opinion which has prevailed respecting its operation. But whether it is received into the circulating, or acts only on the nervous, system; and on what parts of the system it makes its impression in producing each of its effects, can only be determined by experiments made for the purpose. The limits of this Paper will not permit me to enter into a detail of these experiments, for which I must refer to an Essay published some years ago, or a volume which will soon appear, and the works referred to in them; and here confine myself to the general result.

From these experiments it appears, that the effects of opium on living animals may be divided into three classes. Its effects on the brain, on the heart and blood-vessels, and on other parts of the system.

The last do not essentially differ from those of other local irritations. A powerful impression made on the nerves of the stomach is sufficient to destroy life. But opium, although the impression made on the nerves of the stomach by a large dose is very considerable, seems never to occasion death in this way.

The effects of opium on the heart and blood-vessels are that of increasing their action when applied in small quantity; and that of impairing or altogether destroying it, when applied more freely: effects differing in degree but not in kind from those of other agents.* It does not appear that the quantity of opium absorbed by the lacteals from the largest dose, is sufficient to destroy the muscular power of the heart merely by its action on that organ. It may safely be asserted, that opium never kills by destroying the muscular power of the heart, except when a large quantity is injected into it, or into the blood-vessels.—

Opium

* The effects of opium immediately applied to any other muscle are the same.

Opium received into the stomach, therefore, never induces death in this way.

It is ascertained by the experiments alluded to, that the action of the strongest solution of opium, applied to the heart, is merely local; it destroys the excitability of this organ, but it produces no other effect. The excitability of all the other muscles remains unimpaired. It is almost unnecessary to observe, that I here speak of the effects of opium when it is confined to the heart; if it is allowed, in the course of circulation, to pass to the brain, it produces those effects which have been ascribed to its action on the nerves of the heart.

The effects of a small quantity of opium, applied to the brain, are similar to those of other gentle impressions made on this organ, but more powerful, impaired sensibility, languor, sleep. Applied to the brain more freely, it produces effects similar to those of other violent irritations, convulsions and death. And this, it appears, is the way in which opium proves fatal. It is taken up by the lacteals, and, in the course of circulation, applied immediately to the brain. According to the quantity thus applied, it produces sleep, convulsions, or death; for opium, even in the human body, does not always prove fatal when it induces convulsions.

I am, &c.

*Worcester, Jan.* 12, 1804. C. PHILIPS WILSON.

